# Density Functional Theory Analysis of the Copolymerization of Cyclopropenone with Ethylene Using a Palladium Catalyst

**DOI:** 10.3390/polym14235273

**Published:** 2022-12-02

**Authors:** Chenggen Zhang, Shuyuan Yu, Fei Wang, Fuping Wang, Jian Cao, Huimin Zheng, Xiaoyu Chen, Aijin Ren

**Affiliations:** 1 College of Chemistry and Material Science, Langfang Normal University, Langfang 065000, China; 2 College of Chemistry and Chemical Engineering, University of Chinese Academy of Sciences, Beijing 100049, China

**Keywords:** copolymerization, cyclopropenone, reaction mechanism, Pd catalysts, DFT calculations

## Abstract

Density functional theory has been used to elucidate the mechanism of Pd copolymerization of cyclopropenone with ethylene. The results reveal that introducing ethylene and cyclopropenone to Pd catalyst is thermodynamically feasible and generates the α,β-unsaturated ketone unit (UnitA). *Cis*-mode insertion and Path A_1a_ are the most favorable reaction routes for ethylene and cyclopropenone, respectively. Moreover, cyclopropenone decomposition can generate CO in situ without a catalyst or with a Pd catalyst. The Pd-catalyzed decomposition of cyclopropenone exhibits a lower reaction barrier (22.7 kcal/mol) than its direct decomposition. Our study demonstrates that incorporating CO into the Pd catalyst can generate the isolated ketone unit (UnitB). CO is formed first; thereafter, UnitB is generated. Therefore, the total energy barrier of UnitB generation, accounting for the CO barrier, is 22.7 kcal/mol, which is slightly lower than that of UnitA generation (24.0 kcal/mol). Additionally, the possibility of copolymerizing ethylene, cyclopropenone, and allyl acetate (AAc) has been investigated. The free energy and global reactivity index analyses indicate that the cyclopropenone introduction reaction is more favorable than the AAc insertion, which is consistent with the experimental results. Investigating the copolymerization mechanism will help to develop of a functionalization strategy for polyethylene polymers.

## 1. Introduction

Polyolefin materials are extensively being used in various applications. However, the chain structure of olefin polymers is generally saturated because their chains are composed of saturated C-C and C-H bonds. The wide application prospect of polymers is primarily owing to the polar groups on their chains and is determined by their properties. Therefore, it is crucial to introduce highly reactive polar groups into the chains to improve the surface characteristics, adhesion, dyeing and printing properties, solvent resistance, and compatibility and blending with other polymer materials [1,2,3].

Catalysts containing metals are commonly utilized in the copolymerization of olefins and polar monomers to achieve functional modifications. This widely used method can alter the polymer chain structure without applying high temperatures and pressures, significantly increasing the efficiency and yield. Late transition metals, such as Pd and Ni, have been recently proven superior to early transition metals in catalyzing the functionalization of polyolefins.

Since Brookhart et al. first utilized α-diimide Pd and Ni catalyst systems in the polymerization of olefins in 1995 [4], Pd and Ni catalysts have received significant research attention. Compared to other transition metals, Pd and Ni exhibit lower oxygen affinity and improve tolerance to polar functional groups. In 2002, Drent et al. reported another type of revolutionary phosphine sulfonate Ni and Pd catalysts [5] to produce highly linear copolymers of ethylene and acrylate. In functional polyethylene reactions, phosphine sulfonate Pd catalyst is suitable for polar monomers [6], including acrylate [7], acrylic acid [8], vinyl acetate [9], vinyl ether [10], acrylamide [11], and maleic anhydride [12].

In 2012, Guo et al. demonstrated that using a large steric ligand α-diimide Pd catalyst can provide a copolymer with a high degree of polar monomer incorporation [13]. According to Carrow et al., bisphosphine monoxide Pd catalysts produced highly linear copolymers [14,15]. Brookhart et al. showed that the ratio of methyl acrylate in copolymer increases linearly with the increase in methyl acrylate concentration in the presence of a Pd catalyst in 2015 [16]. Sui et al. reported a cationic Pd catalyst, which can copolymerize methyl acrylate and ethylene and generate a copolymer with a high molecular weight [17]. Nozaki et al. developed Pd catalysts based on carbene ligands and propylene copolymers. A series of polar monomers were produced using the Pd catalysts [18], which can copolymerize 1,1-disubstituted ethylene with ethylene [19,20]. In 2016, the Nozaki Group demonstrated that the large, sterically hindered bulky alkyl groups could improve the molecular weight and regioselectivity of the copolymers using phosphine sulfonate Pd catalysts [21]. In 2018, Zhang et al. reported a new cationic phosphonic diamide phosphine Pd catalyst. This catalyst can successfully catalyze ethylene and polar monomers to obtain high-molecular-weight linear copolymers [22]. Chen et al. reported a five-member ring phosphine nitrogen phosphine oxide Pd catalyst. This Pd catalyst can produce copolymers with low molecular weights [23].

The Chen Group recently synthesized a series of functional polyolefins with Pd phosphine sulfonate catalysts and evaluated their performance. They demonstrated that polar polyolefin materials exhibit excellent performance compared to conventional polyolefins [24,25,26]. In 2018, Wang et al. developed chain-end-functionalized polar polyethylenes using a Pd catalyst, introducing a carbene species as the comonomer, and copolymerized ethylene with polar monomers [27].

**Scheme 1:**
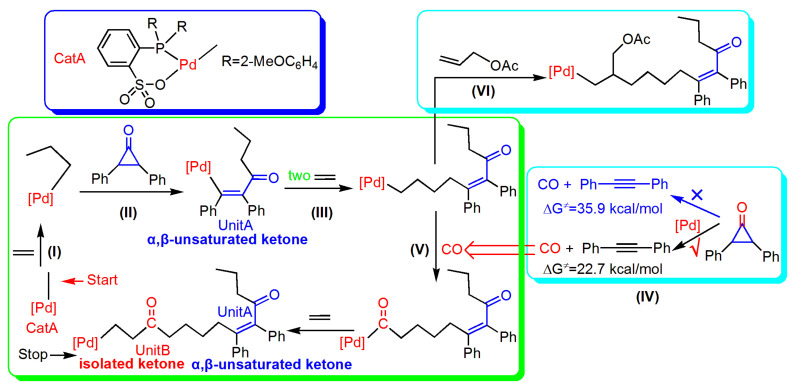
Schematic illustration of Pd-catalyzed copolymerization of ethylene with cyclopropenone mechanism. (**I**) Insterion of the first ethylene into the palladium catalyst (CatA), generates the polymerization product. (**II**) Cyclopropenone is added and the α,β-unsaturated ketone unit (UnitA) is generated. (**III**) The second and third ethylene keep inserting and increase the chain. (**IV**) In-situ generation of CO from cyclopropenone. The palladium-catalyzed decomposition of cyclopropenone has a lower reaction barrier than the direct decomposition. (**V**) Incorporating CO to generate the isolated ketone unit (UnitB). (**VI**) Investigating the possibility of copolymerizing ethylene, cyclopropenone and allyl acetate (AAc).

Phosphine sulfonate Pd catalysts have been confirmed to be tolerant with extensive polar monomers. In addition, Matsuda et al. found that N-Heterocyclic carbene Pd catalysts can catalyze ring opening of diphenylcyclopropenone, and react with phenylacetylene to produce alkenyl alkynyl ketone [28,29]. In 2019, Wang et al. [30] first applied a Pd catalyst (CatA) to catalyze the ring opening copolymerization of cyclopropenone and ethylene, in which cyclopropenone was used as C3 polar monomer [31,32]. They introduced cyclopropenone to the long chain of polyethylene and obtained a copolymer with in-chain α,β-unsaturated ketone units (UnitA) in addition to an isolated ketone (UnitB), which was incorporated into polyethylene. However, the detailed reaction mechanism remains unclear. No theoretical studies have been conducted on the mechanism of the Pd-catalyzed copolymerization of cyclopropenone with ethylene. Therefore, this study employed density functional theory (DFT) calculations to understand the mechanism of the incorporation of UnitA and UnitB into the chain. A detailed mechanistic study of this experimental phenomenon will help understand the copolymerization mechanism and develop a functionalization strategy for olefin polymers. Scheme 1 summarizes the reaction mechanism of the Pd-catalyzed copolymerization of ethylene with cyclopropenone [30].

## 2. Computational Methods

DFT calculations were performed using the Gaussian 16 software [33]. All structures were optimized and identified to be at a minimum (no virtual frequency) or a transition state (TS, with a specific virtual frequency) via frequency analysis at the B3LYP-D3 (BJ) [34,35,36,37]/BSI level. BSI represents a basis set combining the SDD [38] for Pd and 6-31G (d) for non-metal atoms. The pseudo-potential basis set was used for the Pd atom. The energetic results were refined at the M06-2X [39,40]/BSII level via the SMD [41] solvent effects model (toluene as the solvent) using single-point energy calculations. BSII represents a basis set combining SDD for Pd and 6-311++G (d, p) for non-metal atoms. The gas phase B3LYP/BSI harmonic frequency was employed to modify the free energy using heat and entropy at 353.15 K (experimental temperature) and 1 atm pressure, respectively. Notably, the temperature change has negligible effects on the reaction barrier in the calculation of this system. Free energies derived at the M06-2X (SMD, solvent = toluene)/BSII level are discussed in the main text. The NBO charges [42] and Wiberg bond indices were obtained at the B3LYP/BSI level. The reliability of the M06-2X//B3LYP combination is demonstrated by its effective use in addressing various transition metal catalytic reactions [43,44,45,46,47,48,49,50,51,52]. The total energy of all the optimized structures are shown in Table S1.

The cubic files for interaction region indicator (IRI) [53], the global reactivity index [54,55,56,57] and Fukui functions [58,59] analyses were performed using the Multiwfn program 3.8 [60], and the results were visualized by the VMD program 1.9.3 [61]. Additional computational details and detailed data of Fukui functions values for some molecules are shown in Table S2.

## 3. Results and Discussion

### 3.1. First Ethylene Insertion to Phosphine Sulfonate Pd Catalyst

**Figure 1:**
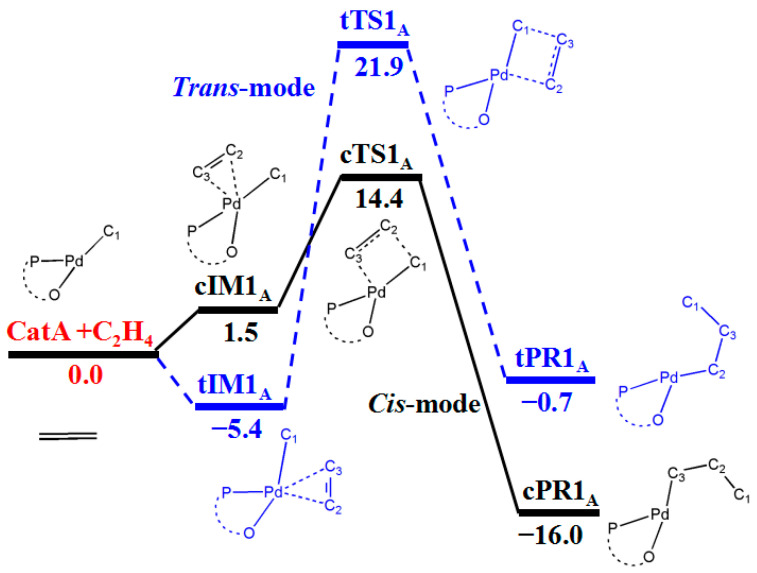
The reaction pathway of the first ethylene insertion, *cis*-mode (black) and *trans*-mode (blue); the relative free energy is given in kcal/mol.

**Figure 2:**
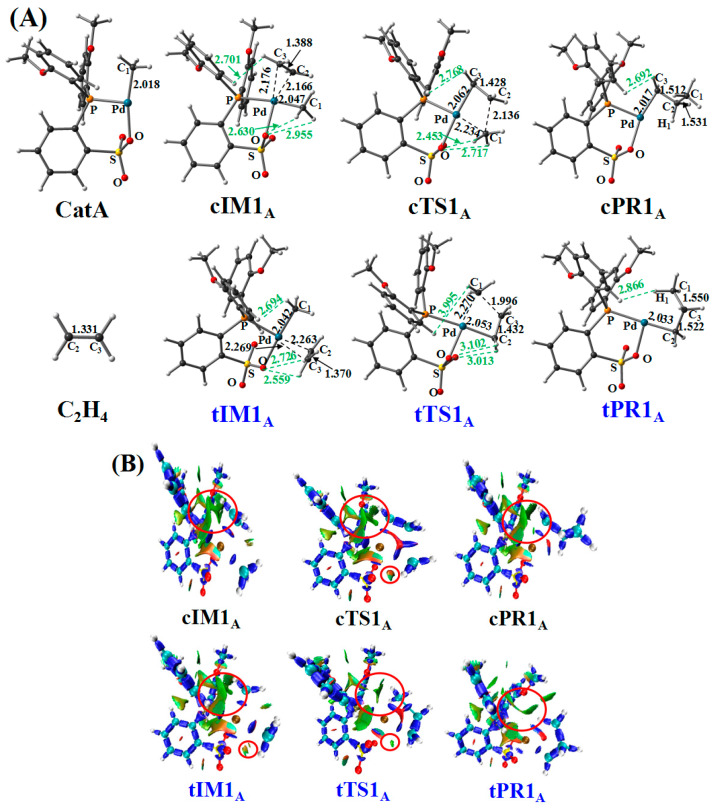
(**A**) Optimized structures in the first ethylene insertion reaction pathway shown in Figure 1, *cis*-mode (black) and *trans*-mode (blue), the key length is in angstroms. (**B**) Interaction region indicator (IRI) analysis of key structures with the noncovalent weak interactions highlighted by the red circles.

We initially investigated the first ethylene insertion to the phosphine sulfonate Pd catalyst (CatA). Figure 1 shows the reaction pathway of the first ethylene insertion and the associated energies. Two possible routes exist for the initial ethylene insertion reaction, the *cis*-mode (ethylene *cis* to phosphorus atom) and *trans*-mode (ethylene *trans* to phosphorus atom) insertion [62]. *Cis*- and *trans*-mode insertion involves the formation of an intermediate (cIM1_A_ or tIM1_A_) followed by a quaternary TS (cTS1_A_ or tTS1_A_), resulting in the final product (cPR1_A_ or tPR1_A_). The main optimized structures are shown in Figure 2.

Pd catalyst (CatA) has two ligands, phosphine and sulfonate. In *cis*-mode insertion, the ethylene first coordinate to the catalyst’s center metal, forming intermediate cIM1_A_ (endothermic; 1.5 kcal/mol). Owing to the coordination effect, in the cIM1_A_ configuration, the Pd-C_1_ bond (2.047 Å) is longer than that in CatA (2.018 Å). Additionally, the C_2_-C_3_ (1.388 Å) bond is longer than that in C_2_H_4_ molecule (1.331 Å). After the formation of the intermediate, cIM1_A_, the reaction reaches the transition state, cTS1_A_ (ΔG = +12.9 kcal/mol), and the Pd-C_1_ (2.234 Å) and C_2_-C_3_ bonds (1.428 Å) are longer. After the transition state, cTS1_A_, the product of the first ethylene insertion, the cPR1_A_ form, is attained (ΔG = −30.4 kcal/mol). Compared to the cTS1_A_ configuration, the C_1_-C_2_ (1.531 Å) and Pd-C_3_ bonds (2.017 Å) in the product cPR1_A_ are shortened by 0.605 and 0.045 Å, respectively. In contrast, the C_2_-C_3_ bond (1.512 Å) in the product cPR1_A_ is elongated by 0.084 Å.

The C_2_-C_3_ bond lengthens during the entire ethylene insertion reaction from a double bond to form a single bond, while the Pd-C_1_ bond lengthens until it breaks. The Wiberg bond indices (WBIs) and natural charges (Q_NBO_) for certain key bonds and atoms involved in the ethylene insertion reaction are presented in Table 1. As the reaction proceeds, the WBIs of the Pd-C_1_ (from 0.704 to 0.010) and C_2_-C_3_ bonds (from 2.039 to 1.069) gradually decrease from the reactant (CatA + C_2_H_4_) to the product cPR1_A_, indicating the breaking of the Pd-C_1_ bond and the C_2_-C_3_ bond change from a double bond to a single bond. The Q_NBO_ values of the C_1_ atom also diminish from −0.819 e in CatA to −0.666 e in the product cPR1_A_. Meanwhile, the WBI gradually increases from 0.408 to 0.672 for the Pd-C_3_ bond and from 0.538 to 1.108 for the C_1_-C_2_ bond, indicating the formation of the Pd-C_3_ and C_1_-C_2_ bonds. The *cis*-mode ethylene insertion reaction is exothermic (ΔG = −16.0 kcal/mol), and the barrier is low at 14.4 kcal/mol, suggesting that the experiment should be simple to perform.

The process of *trans*-mode ethylene insertion is similar to that of the *cis*-mode insertion. The energy results indicate that *cis*-mode insertion is thermodynamically and kinetically preferred to *trans*-mode insertion. Although the energy of cIM1_A_ is 6.9 kcal/mol higher than tIM1_A_, meanwhile, the energies of cTS1_A_ and cPR1_A_ are 7.5 and 15.3 kcal/mol lower than tTS1_A_ and tPR1_A_, respectively. This energy difference can be partly attributed to noncovalent weak interactions. As shown in Figure 2A, the hydrogen bonds in tIM1_A_ (2.559, 2.726, and 2.694 Å) are shorter than those in cIM1_A_ (2.630, 2.955, and 2.701 Å). However, the hydrogen bonds in tTS1_A_ (3.102, 3.013, and 3.995 Å) are longer than those in cTS1_A_ (2.453, 2.717, and 2.768 Å); the hydrogen bond in tPR1_A_ (2.866 Å) is longer than that in cPR1_A_ (2.692 Å). Figure 2B shows the interaction region indicator (IRI) analysis of key structures with weak key interactions highlighted using red circles [53]. As shown in Figure 2B, the noncovalent weak interactions in tIM1_A_ are stronger than the interactions in cIM1_A_. However, the weak interactions in tTS1_A_ and tPR1_A_ are weaker than those in cTS1_A_ and cPR1_A_. Consequently, *cis*-mode insertion is preferred to *trans*-mode insertion during the entire reaction, which is consistent with the previous results demonstrated by Sun [63] and Nozaki [64]. Therefore, only the favorable *cis*-mode ethylene insertion was considered in this following study.

### 3.2. Reaction of Cyclopropenone

**Figure 3:**
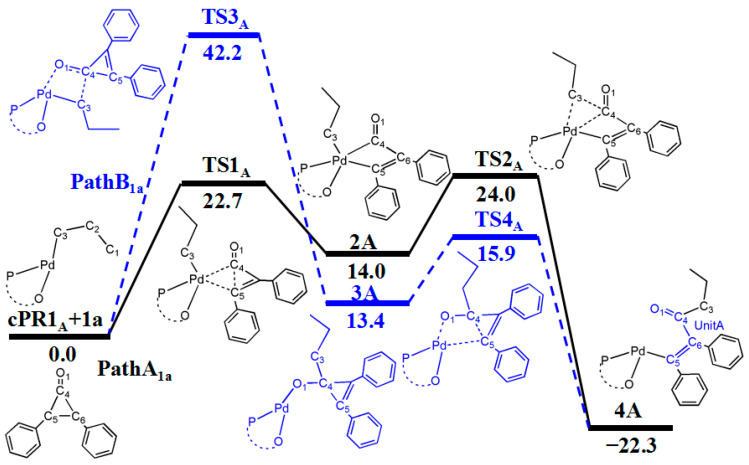
The reaction pathway of cyclopropenone, Path A_1a_ (black) and Path B_1a_ (blue); the relative free energy is given in kcal/mol.

**Figure 4:**
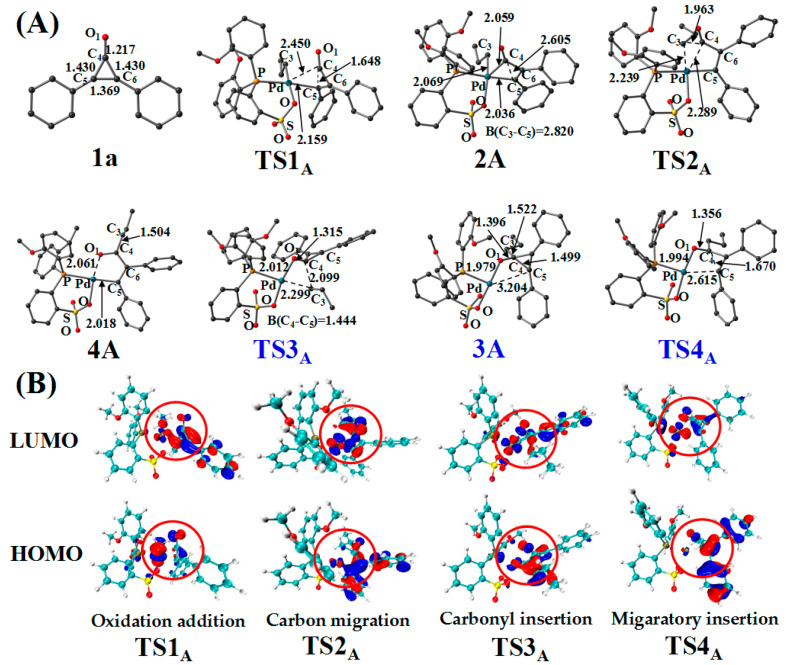
(**A**) Optimized structures in the cyclopropenone reaction pathway shown in Figure 3, Path A_1a_ (black) and Path B_1a_ (blue), the key length is in angstroms. For clarity, the hydrogen atom is omitted. (**B**) The frontier orbital (HOMO and LUMO) of the transition states.

After the ethylene insertion into the catalyst, the subsequent reaction of cyclopropenone (1a) could occur via one of two paths. Path A_1a_ and Path B_1a_ result in the same product (4A), by first forming a transition state (TS1_A_ or TS3_A_) and then an intermediate (2A or 3A), followed by the formation of another TS (TS2_A_ or TS4_A_). Figure 3 illustrates the reaction pathway of cyclopropenone, and Figure 4 shows the main optimized structures for the cyclopropenone reaction.

In Path A_1a_, the C_4_-C_5_ bond of cyclopropenone first coordinates to the Pd metal of cPR1_A_, forming a three-member oxidative addition transition state, TS1_A_ (ΔG = +22.7 kcal/mol). The length (1.648 Å) of the C_4_-C_5_ bond in the configuration of the transition state TS1_A_ is longer than that of 1a (1.430 Å) by 0.218 Å. Subsequently, the intermediate 2A is formed (ΔG = −8.7 kcal/mol), completing the oxidative addition process. Compared to the TS1_A_, in the 2A configuration, the C_4_-C_5_ (2.605 Å) bond is longer than that (1.648 Å) in the TS1_A_. The Pd-C_4_ (2.059 Å) and Pd-C_5_ (2.036 Å) bonds are shorter than those in the TS1_A_. Thereafter, the intermediate, 2A, evolves to another three-member carbon migration transition state, TS2_A_ (ΔG = +10.0 kcal/mol). Τhe Pd-C_3_ (2.239 Å) and Pd-C_4_ bonds (2.289 Å) become longer. Afterward, the Pd-C_3_ bond breakage leads to the product, 4A, which contains UnitA (ΔG = −46.3 kcal/mol). The C_3_-C_4_ bond (1.504 Å) of 4A is shorter than that in the TS2_A_ (1.963 Å) configuration by 0.459 Å. The highest occupied orbital (HOMO) and the lowest unoccupied orbital (LUMO) of TS1_A_ and TS2_A_ also indicate their oxidative addition and carbon migration reaction characteristics, respectively. The WBI and Q_NBO_ values for certain key bonds and atoms involved in the cyclopropenone reaction are listed in Table 2. As the reaction Path A_1a_ proceeds from TS1_A_ to the product 4A, the WBI decreases from 0.691 to 0.017 for the Pd-C_3_ bond and from 0.730 to 0.061 for the C_4_-C_5_ bond. This decrease indicates the breakage of Pd-C_3_ and C_4_-C_5_ bonds. From the TS1_A_ to the 4A, the WBI progressively increases from 0.298 to 0.685 for the Pd-C_5_ bond and from 0.025 to 1.008 for the C_3_-C_4_ bond, indicating the formation of these bonds. The free energy of TS2_A_ is slightly higher than TS1_A_ by 1.3 kcal/mol. Compared to the reactant (cPR1_A_ + 1a), the energy barrier for Path A_1a_ is 24.0 kcal/mol, and the reaction is exothermic (ΔG = −22.3 kcal/mol), implying that it is thermodynamically feasible.

In Path B_1a_ of the cyclopropenone reaction, the C_4_-O_1_ bond of cyclopropenone is first inserted into the Pd-C_3_ bond of cPR1_A_, forming a four-member carbonyl insertion transition state TS3_A_ (ΔG = +42.2 kcal/mol). The lengths of Pd-C_3_ (2.299 Å) and C_4_-O_1_ (1.315 Å) bonds in the TS3_A_ configuration are longer than those (2.017 and 1.217 Å) in the reactant (cPR1_A_ + 1a). Thereafter, the intermediate 3A is formed (ΔG = −28.8 kcal/mol). In the 3A configuration, the C_4_-O_1_ (1.396 Å) bond and C_4_-C_5_ (1.499 Å) bond are longer than those (1.315 Å and 1.444 Å) in TS3_A_. The Pd-O_1_ (1.979 Å) bond is shorter than that (2.012 Å) in TS3_A_. Afterward, the intermediate, 3A, evolves to another four-member migratory insertion TS, TS4_A_ (ΔG = +2.5 kcal/mol). Meanwhile, the Pd-O_1_ (1.994 Å) and C_4_-C_5_ bonds (1.670 Å) elongate. Afterward, the C_4_-C_5_ bond breakage leads to the product, 4A (ΔG = −38.2 kcal/mol). Compared to the TS4_A_ configuration, the Pd-C_5_ bond (2.018 Å) of 4A is shorter than that in the TS4_A_ (2.615 Å) configuration by 0.597 Å. The frontier orbitals (HOMO and LUMO) of the transition states TS3_A_ and TS4_A_ show their respective carbonyl insertion and migratory insertion reaction characteristics.

The energy of TS3_A_ is higher than that of TS4_A_ by 26.3 kcal/mol. Compared to the reactant (cPR1_A_ + 1a), the energy barrier for Path B_1a_ is 42.2 kcal/mol, which is higher than that for Path A_1a_ (24.0 kcal/mol). Consequently, Path A_1a_ is a more favorable reaction route than Path B_1a_. The barrier for cyclopropenone Path A_1a_ is 9.6 kcal/mol higher than that of the initial ethylene insertion reaction. However, the reaction for cyclopropenone Path A_1a_ is 6.3 kcal/mol more exothermic than ethylene insertion.

### 3.3. Second and Third Ethylene Insertion

**Figure 5:**
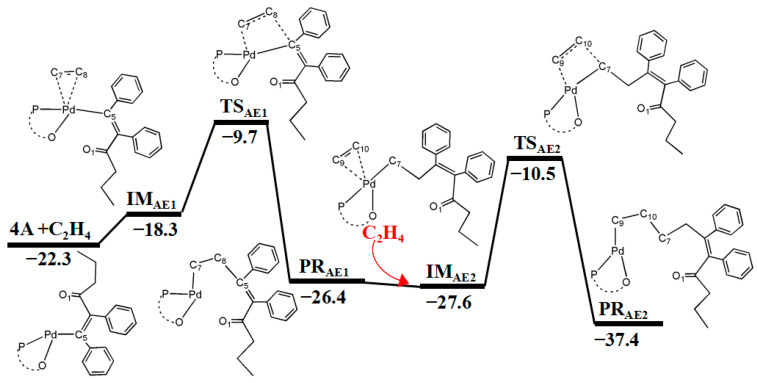
The reaction pathway for continuing ethylene insertion; the relative free energy is given in kcal/mol.

**Figure 6:**
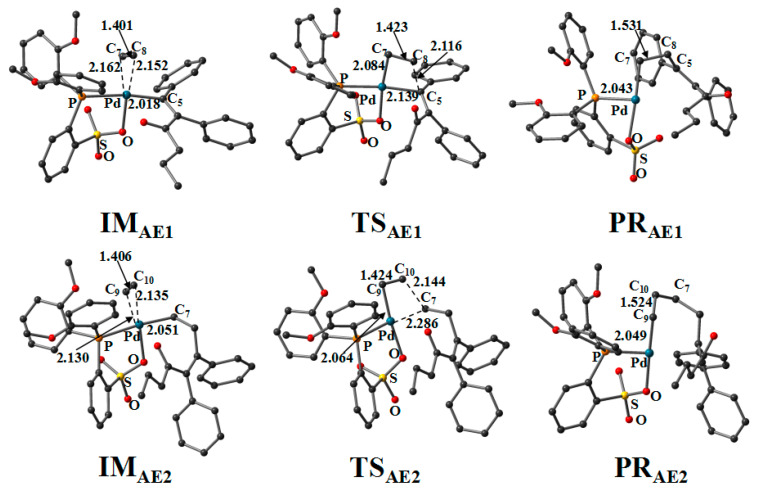
Optimized structures for continuing ethylene insertion reaction pathway shown in Figure 5, the key length is in angstroms. For clarity, the hydrogen atom is omitted.

After introducing cyclopropenone into the reaction chain, ethylene insertion into the chain occurs continuously. We explored the reaction mechanism by performing the second and third ethylene insertion. Figure 5 illustrates the pathway for continuous ethylene insertion, while Figure 6 shows the main optimized structures of this reaction.

For the second ethylene insertion, 4A and C_2_H_4_ first form a coordination intermediate IM_AE1_ (ΔG = +4.0 kcal/mol). In the IM_AE1_ configuration, Pd-C_7_ and Pd-C_8_ bond lengths are 2.162 and 2.152 Å, respectively. After the formation of the intermediate IM_AE1_, the reaction reaches the transition state TS_AE1_ (ΔG = +8.6 kcal/mol). Τhe Pd-C_5_ (2.139 Å) and C_7_-C_8_ bonds (1.423 Å) become longer, while the Pd-C_7_ bond (2.084 Å) becomes shorter. After the TS_AE1_, the product PR_AE1_ is formed (ΔG = −16.7 kcal/mol). The C_7_-C_8_ bond (1.531 Å) in the product PR_AE1_ is 0.108 Å longer than that in the TS_AE1_ configuration. The energy barrier for the pathway of the second ethylene insertion is 12.6 kcal/mol, and the reaction is exothermic (ΔG = −4.1 kcal/mol), implying that the reaction is feasible.

Similar to the second ethylene insertion, in the reaction pathway for the third ethylene insertion, an intermediate (IM_AE2_) is first formed, followed by a quaternary TS (TS_AE2_), finally resulting in the product (PR_AE2_). The barrier for the third ethylene insertion is 15.9 kcal/mol, which is slightly higher than that of the second ethylene insertion (12.6 kcal/mol). The reaction is exothermic (ΔG = −11.0 kcal/mol), releasing more energy than the second ethylene insertion (ΔG = −4.1 kcal/mol), which indicates that the reaction is feasible.

### 3.4. Generation of CO from Cyclopropenone

**Figure 7:**
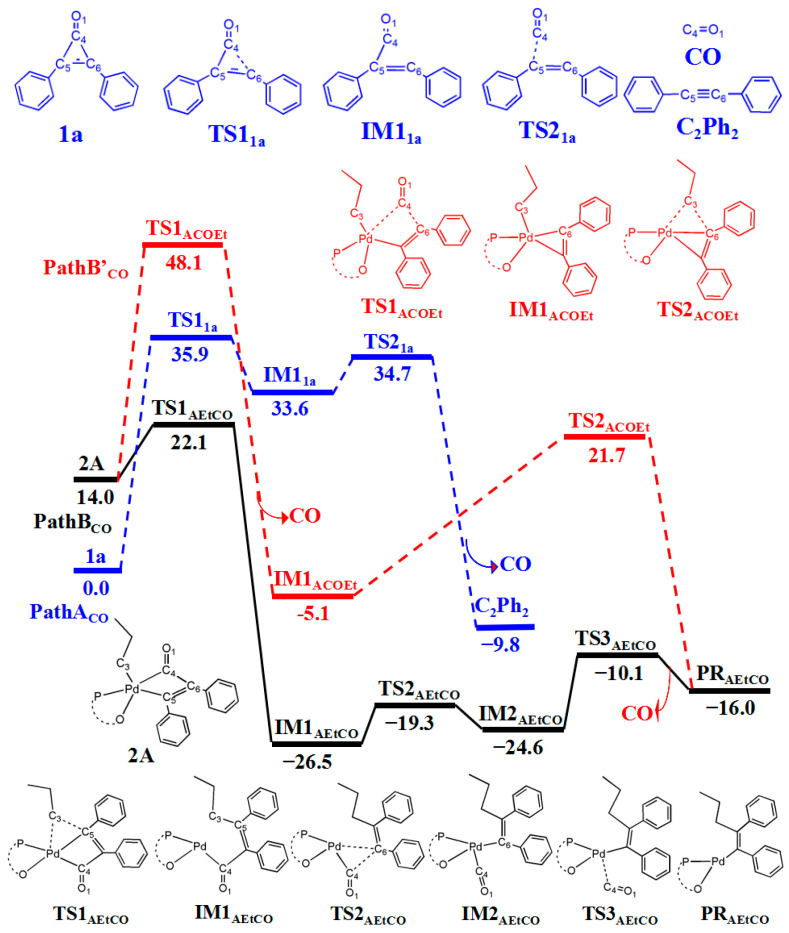
Generation CO from cyclopropenone decomposition reaction pathway without catalyst (Path A_CO_, blue) or with pd catalyst (Path B_CO_, black; Path B’_CO_, red); the relative free energy is given in kcal/mol.

**Figure 8:**
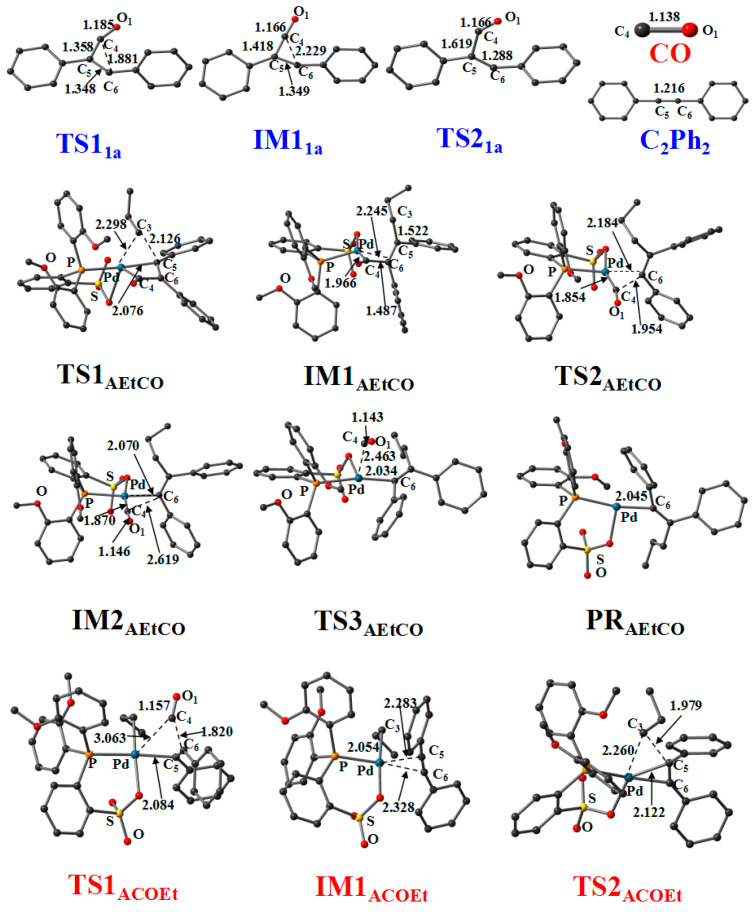
Optimized structures in the pathway of generation CO from cyclopropenone shown in Figure 7 (Path A_CO_, blue; Path B_CO_, black; Path B’_CO_, red), the key length is in angstroms. For clarity, the hydrogen atom is omitted.

Cyclopropenone can break down into CO and alkynes [65,66]. This study investigated the decomposition of cyclopropenone without a catalyst (Path A_CO_) and with a Pd catalyst (Path B_CO_; Path B’_CO_). Figure 7 illustrates the pathway of generation of CO from cyclopropenone. The main optimized structures involved in the generation of CO from cyclopropenone decomposition are shown in Figure 8.

In the Path A_CO_, cyclopropenone (1a) decomposes without a catalyst. The reaction first forms a transition state (TS1_1a_). The C_4_-C_6_ bond (1.881 Å) is longer than that of 1a (1.430 Å), whereas the C_4_-O_1_ bond length has decreased from 1.217 Å in 1a to 1.185 Å in the TS1_1a_ configuration. Relative to 1a, the barrier for TS1_1a_ is 35.9 kcal/mol. Subsequently, the intermediate IM1_1a_ is formed through an exothermic reaction with ΔG = −2.3 kcal/mol, relative to TS1_1a_. The C_4_-C_6_ (2.229 Å) and C_4_-C_5_ (1.418 Å) bonds in the IM1_1a_ configuration are longer than those in the TS1_1a_, whereas the C_4_-O_1_ (1.166 Å) bond is shorter. IM1_1a_ is followed by the formation of another transition state, TS2_1a_, across a small barrier of 1.1 kcal/mol. The C_4_-C_5_ bond (1.619 Å) increases in length. Afterward, the C_4_-C_5_ bond breakage leads to the product CO + C_2_Ph_2_, through an exothermic reaction (ΔG = −44.5 kcal/mol). Τhe C_4_-O_1_ bond (1.138 Å) of CO is 0.028 Å shorter than that in the TS2_1a_ configuration.

In Path A_CO_, the C_4_-C_6_ bond lengths gradually increase until they break, while the C_4_-O_1_ bond lengths gradually decrease until they form a free CO molecule. WBI and Q_NBO_ values for certain key bonds and atoms involved in the generation of CO from cyclopropenone are listed in Table 3. As the reaction proceeds from the reactant (1a) to TS2_1a_, the WBIs gradually decrease, from 1.078 to 0.806 for the C_4_-C_5_ bond and from 1.078 to 0.391 for the C_4_-C_6_ bond, indicating the breakage of these bonds. Meanwhile, the WBIs of the C_4_-O_1_ (from 1.682 to 2.250) and the C_5_-C_6_ (from 1.478 to 2.660) bonds gradually increase from 1a to the product (CO + C_2_Ph_2_), indicating that the decomposition of cyclopropenone form free CO and C_2_Ph_2_. Although Path A_CO_ is an exothermic reaction (ΔG = −9.8 kcal/mol), the barrier is very high (35.9 kcal/mol), implying that the reaction is laborious.

In Path B_CO_, cyclopropenone (1a) decomposes with a Pd catalyst (CatA). An intermediate (2A) initially forms in the cyclopropenone reaction pathway, as shown in Figure 3. The reaction first forms a three-member alkyl (C_3_) transfer transition state (TS1_AEtCO_). The Pd-C_3_ (2.298 Å) bond is longer in the TS1_AEtCO_ configuration than that in 2A (2.069 Å), and the C_3_-C_5_ bond length decreases from 2.820 (in 2A) to 2.126 Å. Relative to 2A, the barrier for TS1_AEtCO_ is 8.1 kcal/mol. Thereafter, the intermediate IM1_AEtCO_ forms through an exothermic reaction (ΔG = −48.6 kcal/mol). In the IM1_AEtCO_ configuration, the C_3_-C_5_ (1.522 Å) bond is significantly shorter than that in the TS1_AEtCO_ (2.126 Å). Afterward, the Pd-C_3_ bond breaks. The intermediate IM1_AEtCO_ is followed by the endothermic formation of another carbon (C_6_) transfer transition state, TS2_AEtCO_ (ΔG = +7.2 kcal/mol). Τhe C_4_-C_6_ bond length is 1.954 Å, which is longer than that in IM1_AEtCO_ (1.487 Å). After TS2_AEtCO_, the C_4_-C_6_ bond breaks leading to IM2_AEtCO_ (ΔG = −5.3 kcal/mol). In the IM2_AEtCO_ configuration, the C_4_-C_6_ (2.619 Å) bond is considerably longer than that in TS2_AEtCO_ (1.954 Å). The intermediate IM2_AEtCO_ is followed by the formation of the transition state TS3_AEtCO_ (ΔG = +14.5 kcal/mol), and the Pd-C_4_ bond (2.463 Å) becomes longer than that in IM2_AEtCO_ (1.870 Å). Thereafter, the reaction releases CO molecules and produces CO + PR_AEtCO_, through an exothermic reaction (ΔG = −5.9 kcal/mol).

In Path B_CO_, the Pd-C_3_ and Pd-C_5_ bonds gradually elongates until their breakage, and the Pd-C_6_ bonds gradually shortens until they form stable bonds. From 2A to the intermediate IM2_AEtCO,_ the WBI of the Pd-C_3_ bond decreases from 0.629 to 0.020 and that of the Pd-C_5_ bond decreases from 0.564 to 0.031, indicating the breakage of these bonds. Meanwhile, the WBI of the Pd-C_6_ bond increases from 0.080 in 2A to 0.605 in IM2_AEtCO_, indicating the formation of the Pd-C_6_ bond. The Q_NBO_ value of the C_6_ atom also gradually increases from −0.184 e in 2A to −0.091 e in the intermediate IM2_AEtCO_.

Because 2A is an intermediate of the cyclopropenone reaction pathway (Figure 3), the energy of the reactant (cPR1_A_ + 1a) and the first transition state (TS1_A_) for the cyclopropenone reaction should be considered when analyzing the barrier and exothermic conditions of Path B_CO_. Compared to the reactant of cyclopropenone reaction (cPR1_A_ + 1a), the reaction Path B_CO_ is an exothermic reaction (ΔG = −16.0 kcal/mol). The barrier of TS1_A_ (22.7 kcal/mol) is 0.6 kcal/mol higher than that of TS1_AEtCO_ (22.1 kcal/mol). The energy barrier of Path B_CO_ is 22.7 kcal/mol, which is significantly lower than that of Path A_CO_ (35.9 kcal/mol), indicating that Path B_CO_ is favorable, and the reaction is feasible.

Path B’_CO_ is another pathway for cyclopropenone (1a) to decompose with a Pd catalyst (CatA). Path B’_CO_ begins from the intermediate (2A) (Figure 3) of the cyclopropenone reaction pathway. In Path B’_CO_, 2A first forms a transition state (TS1_ACOEt_) and then releases CO molecules to the intermediate (IM1_ACOEt_). Next, another alkyl (C_3_) transfer TS (TS2_ACOEt_) is formed, resulting in the same product with Path B_CO_ (PR_AEtCO_). The main difference between Path B’_CO_ and Path B_CO_ is that in Path B’_CO_, CO is first released, and then, the alkyl transfer occurs to afford the product, while in Path B_CO,_ CO is released after the alkyl transfer. Compared with the reactant of cyclopropenone reaction (cPR1_A_ + 1a), the barrier of the reaction Path B’_CO_ is 48.1 kcal/mol, which is higher than that of Path B_CO_ (22.7 kcal/mol), implying that Path B’_CO_ is not feasible.

### 3.5. Reaction Pathway for the Generation of UnitB

**Figure 9:**
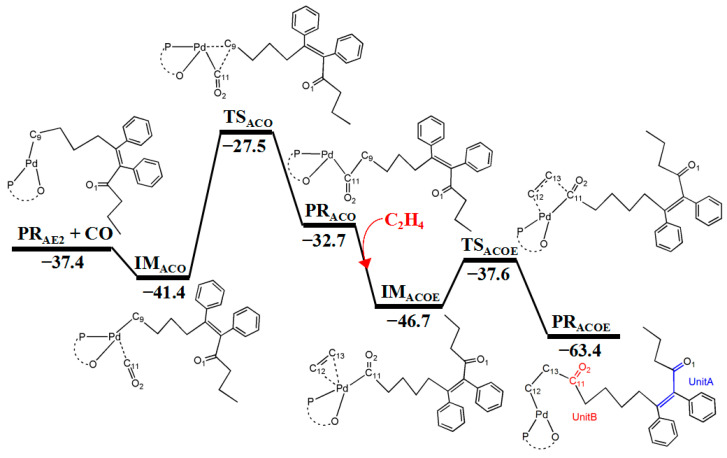
The reaction pathway for generation of UnitB; the relative free energy is given in kcal/mol.

**Figure 10:**
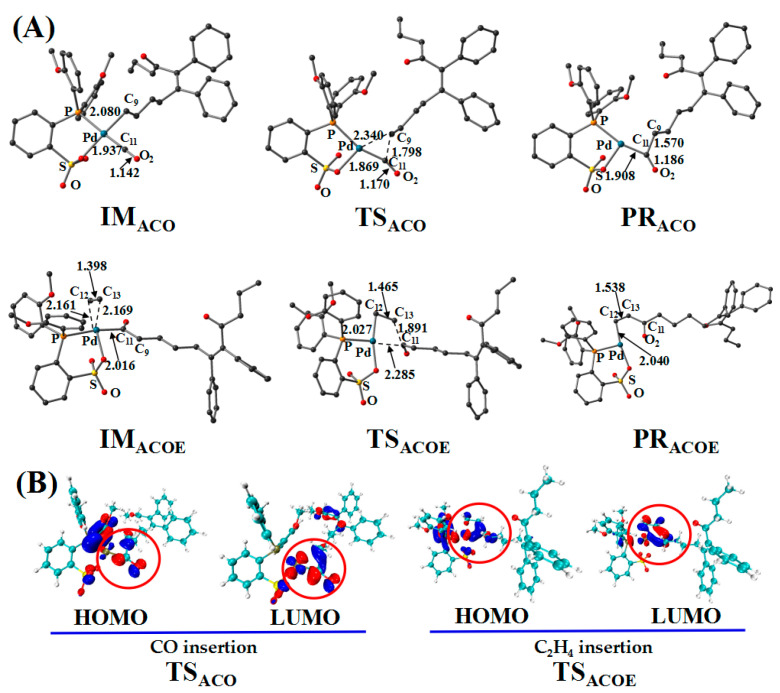
(**A**) Optimized structures in the reaction pathway for generation of UnitB shown in Figure 9, the key length is in angstroms. For clarity, the hydrogen atom is omitted. (**B**) The frontier orbital (HOMO and LUMO) of the transition states.

PR_AE2_, the product obtained after the second and third ethylene insertions (Figure 5), can continue reacting with CO to form UnitB. Figure 9 shows the reaction pathway for the generation of UnitB. The main optimized structures for the reaction pathway for the generation of UnitB are shown in Figure 10. Table 4 lists the WBI and Q_NBO_ values for certain key bonds and atoms of the reaction pathway for the generation of UnitB.

The reaction pathway for the generation of UnitB can be divided into two parts, the CO insertion reaction and the ethylene insertion reaction. In the CO insertion reaction, the carbon atom (C_11_) in CO is first coordinated with the Pd metal atom of the PR_AE2_, forming the intermediate IM_ACO_ (ΔG = −4.0 kcal/mol). Owing to the coordination effect, the Pd-C_9_ bond (2.080 Å) in the IM_ACO_ configuration is longer than that in PR_AE2_ (2.049 Å). The Q_NBO_ value of the Pd atom decreases from 0.232 e in PR_AE2_ to 0.026 e in the intermediate IM_ACO_. Meanwhile, the Q_NBO_ values of the C_11_ (0.665 e) and O_2_ (−0.415 e) atoms in the intermediate IM_ACO_ are higher than those in PR_AE2_ (0.506 e and −0.506 e). After the formation of IM_ACO_, the reaction reaches the three-member transition state, TS_ACO_ (ΔG = +13.9 kcal/mol). Τhe Pd-C_9_ (2.340 Å) and C_11_-O_2_ bonds (1.170 Å) become longer, whereas the Pd-C_11_ bond (1.869 Å) becomes shorter. As shown in Figure 10B, the characteristics of the CO insertion reaction can be determined from the frontier orbitals (HOMO and LUMO) of TS_ACO_. After TS_ACO_, the product of the CO insertion, PR_ACO,_ forms through an exothermic reaction (ΔG = −5.2 kcal/mol). The C_9_-C_11_ bond length in PR_ACO_ is 1.570 Å, which is 0.228 Å smaller than that in the TS_ACO_ configuration. Additionally, the Pd-C_11_ (1.908 Å) and C_11_-O_2_ bonds (1.186 Å) in PR_ACO_ are longer by 0.039 and 0.016 Å, respectively. As the CO insertion reaction proceeds, the WBI of the Pd-C_9_ bond decreases from 0.701 in the intermediate IM_ACO_ to 0.158 in the product PR_ACO_, indicating the Pd-C_9_ bond breakage. Meanwhile, the WBI of the C_9_-C_11_ bond increases from 0.076 in the intermediate IM_ACO_ to 0.919 in the product PR_ACO_, indicating the formation of the C_9_-C_11_ bond. The CO insertion reaction is endothermic (ΔG = +4.7 kcal/mol), with a barrier of only 9.9 kcal/mol, indicating that the CO insertion reaction is feasible.

For the ethylene insertion reaction, the ethylene is first coordinated to the Pd metal atom in PR_ACO_, forming the intermediate IM_ACOE_ (ΔG = −14.0 kcal/mol). Owing to the coordination effect, the Pd-C_11_ bond in the IM_ACOE_ configuration is 2.016 Å and is longer than that in PR_ACO_ (1.908 Å). After the formation of the intermediate IM_ACOE_, the reaction reaches the four-member transition state, TS_ACOE_ (ΔG = +9.1 kcal/mol). The Pd-C_11_ (2.285 Å) and C_12_-C_13_ bonds (1.465 Å) become longer, while the C_13_-C_11_ bond (1.891 Å) becomes shorter. As shown in Figure 10B, the characteristics of the C_2_H_4_ insertion reaction can be determined from the frontier orbitals (HOMO and LUMO) of TS_ACOE_. After the TS_ACOE_, the product of the ethylene insertion, PR_ACOE_, is formed through an exothermic reaction (ΔG = −25.8 kcal/mol). PR_ACOE_ contains UnitB. Compared to the TS_ACOE_ configuration, the C_12_-C_13_ bond (1.538 Å) in PR_ACOE_ is elongated by 0.073 Å. As the ethylene insertion reaction proceeds, the WBI of the Pd-C_11_ bond decreases from 0.748 in PR_ACO_ to 0.022 in PR_ACOE_ and that of the C_12_-C_13_ bond decreases from 2.039 to 1.010, indicating the breakage of the Pd-C_11_ bond, and the C_12_-C_13_ bond changes from a double bond to a single bond. Meanwhile, the WBIs of the Pd-C_12_ (from 0.411 to 0.705) and C_11_-C_13_ (from 0.051 to 1.026) bonds gradually increases from the intermediate IM_ACOE_ to the product PR_ACOE_, indicating the formation of the Pd-C_12_ and C_11_-C_13_ bonds. The ethylene insertion reaction is exothermic (ΔG = −30.7 kcal/mol), and the barrier is very low, indicating that the ethylene insertion reaction is feasible. In the reaction pathway for the generation of UnitB, the free energy of TS_ACO_ is the highest. Compared to the free energy of the reactant (PR_AE2_ + CO), the reaction pathway for the generation of UnitB is exothermic (ΔG = −26.0 kcal/mol), and the barrier is only 9.9 kcal/mol, suggesting that the reaction is viable.

For producing UnitB, CO should be formed first, implying that CO generation should be considered in the determination of the total energy barrier of UnitB. The energy barrier of CO generation is 22.7 kcal/mol (i.e., the energy of TS1_A_, as shown in Figure 3), which is higher than 9.9 kcal/mol. Therefore, the total energy barrier for UnitB generation is 22.7 kcal/mol, which is slightly lower than that for UnitA generation (24.0 kcal/mol), as shown in Figure 3. However, CO gas is generated in situ, and not all the generated CO can be smoothly inserted into the polymerization chain. Consequently, the UnitB generation in the polymerization chain increases only when the CO generation increases.

### 3.6. Reaction Pathway of the Allyl Acetate (AAc) Insertion

**Figure 11:**
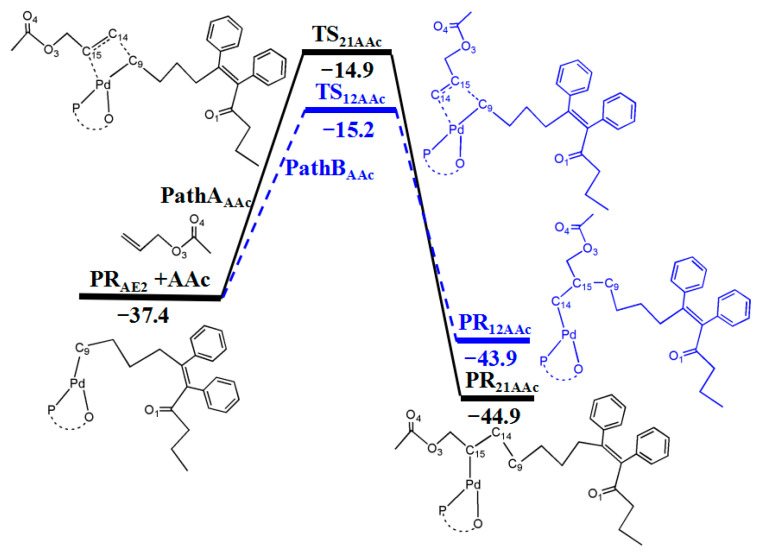
The reaction pathway of the allyl acetate (AAc) insertion, Path A_AAc_ (black) and Path B_AAc_ (blue); the relative free energy is given in kcal/mol.

**Figure 12:**
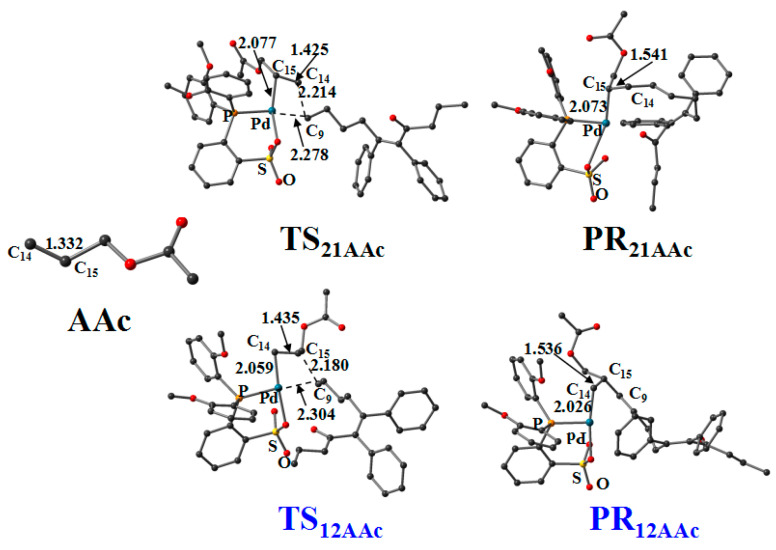
Optimized structures in the allyl acetate (AAc) insertion reaction pathway shown in Figure 11, Path A_AAc_ (black) and Path B_AAc_ (blue), the key length is in angstroms. For clarity, the hydrogen atom is omitted.

To examine the possibility of the copolymerization of ethylene, cyclopropenone, and AAc, we investigated the reaction mechanism of introducing AAc into PR_AE2_. Figure 11 illustrates the reaction pathway of the AAc insertion. Figure 12 shows the main optimized structures for the reaction pathway of the AAc insertion. The WBI and Q_NBO_ values for certain key bonds and atoms of the reaction pathway of the AAc insertion are listed in Table 5.

The AAc insertion reaction could be via Path A_AAc_ or Path B_AAc_. In Path A_AAc_, AAc and PR_AE2_ first form a quaternary transition state TS_21AAc_ (ΔG = +22.5 kcal/mol). Τhe Pd-C_9_ (2.278 Å) and C_14_-C_15_ bonds (1.425 Å) become longer. Afterward, the product of the AAc insertion, PR_21AAc,_ is formed (ΔG = −30.0 kcal/mol). Compared to the TS_21AAc_ configuration, the C_14_-C_15_ bond (1.541 Å) in the product PR_21AAc_ is lengthened by 0.116 Å. As the reaction proceeds, the WBI of the Pd-C_9_ bond decreases from 0.706 to 0.066, indicating the breakage of the Pd-C_9_ bond. The WBI of the C_14_-C_15_ bond decreases from 2.039 to 1.014, implying the transition from a double bond to single bond. Meanwhile, the WBIs of the Pd-C_15_ (from 0.518 to 0.663) and C_9_-C_14_ (from 0.487 to 1.004) bonds gradually increase from TS_21AAc_ to PR_21AAc_, indicating the formation of the Pd-C_15_ and C_9_-C_14_ bonds. Path A_AAc_ is an exothermic reaction (ΔG = −7.5 kcal/mol) with a barrier of 22.5 kcal/mol, implying that the reaction is feasible. Similar to Path A_AAc_, in Path B_AAc_, a quaternary transition state (TS_12AAc_) is first formed, resulting in the product (PR_12AAc_). Path B_AAc_ is an exothermic reaction (ΔG = −6.5 kcal/mol) with an energy barrier of 22.2 kcal/mol, indicating that Path A_AAc_ and Path B_AAc_ compete with each other.

The reaction barrier of the cyclopropenone introduction is 24.0 kcal/mol (Figure 3), which is slightly higher than that of the AAc insertion reaction (22.5 or 22.2 kcal/mol). However, the exothermic cyclopropenone reaction (ΔG = −22.3 kcal/mol) releases significantly more energy than the AAc insertion reaction (ΔG = −7.5 or −6.5 kcal/mol). These findings indicate that the cyclopropenone introduction reaction is more likely to occur, which is consistent with the experimental results [30].

To further investigate the reactions, we calculated the global reactivity index (GRI) and Fukui function values for certain molecules (Table 6). Compared to the other three molecules, the catalyst (CatA) has the largest electrophilicity (*ω*) value of 1.473, indicating that it is electrophilic. Meanwhile, CatA has a large global nucleophilicity (*N*_Nu_) value of 3.105, indicating that it is also nucleophilic. The *N*_Nu_ value of 1a (3.283) is higher than that of AAc (1.972); therefore, the electrophilicity of 1a is higher, and it exhibits higher reactivity. This is consistent with the experimental results [30].

**Figure 13:**
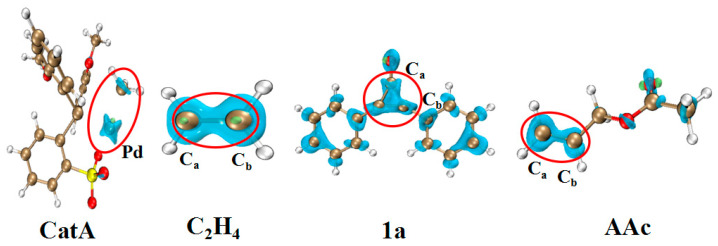
Isosurface maps of Fukui functions for Pd (*f^+^*) in CatA and C_a_, C_b_ (*f^−^*) in C_2_H_4_, 1a and AAc.

The Fukui function is often employed to predict reactions. The site with a higher Fukui function value has a higher reactivity. The Fukui function information for certain molecules is presented in Table 6 and Figure 13. The complete Fukui function values of these molecules are provided in Table S2. The results show that the Fukui function value (*f^+^*) of the Pd atom is the most significant parameter of CatA; therefore, the Pd atom in CatA is more reactive. The Fukui function values (*f^−^*) of C_a_ and C_b_ atoms in C_2_H_4_ are the highest. Meanwhile, the Fukui function values (*f^−^*) of C_a_ and C_b_ atoms in 1a and AAc, respectively, are the highest except for the oxygen atom. This indicates that C_a_ and C_b_ are more reactive, easily reacting with Pd in CatA to complete the reactions.

## 4. Conclusions

We performed DFT calculations to investigate the mechanism of Pd-catalyzed copolymerization of cyclopropenone with ethylene. The results demonstrated that introducing ethylene and cyclopropenone to Pd catalyst is thermodynamically feasible and generated UnitA. *Cis*-mode insertion and Path A_1a_ is the most favorable reaction route for ethylene and cyclopropenone, respectively. The energy barrier for cyclopropenone Path A_1a_ is higher than that for the ethylene insertion reaction. However, more energy (6.3 kcal/mol) is released in the cyclopropenone exothermic reaction than in the ethylene insertion. Additionally, cyclopropenone can decompose to generate CO in situ without a catalyst or with a Pd catalyst. The Pd-catalyzed decomposition of cyclopropenone has a lower reaction barrier than the direct decomposition of cyclopropenone. The energy barrier of CO generation is 22.7 kcal/mol. Incorporating CO into a Pd catalyst can generate UnitB. CO should be formed first, and then, UnitB is generated. Therefore, the total energy barrier of UnitB generation should be determined by considering the energy barrier of CO generation. The energy barrier of incorporating CO to a Pd catalyst is 9.9 kcal/mol, which is lower than that of CO generation. Therefore, the total energy barrier for UnitB generation is 22.7 kcal/mol, which is slightly lower than that for UnitA generation (24.0 kcal/mol). Moreover, the possibility of copolymerizing ethylene, cyclopropenone, and AAc was investigated. The free energy and the GRI analyses indicate that the cyclopropenone insertion reaction is more favorable than AAc insertion, which is consistent with the experimental results. A thorough mechanistic study of this phenomenon will contribute to understanding the copolymerization of cyclopropenone with ethylene and developing a functionalization strategy for PE polymers.

## Figures and Tables

**Table 1:** The Wiberg bond indices (WBIs) and natural charges (Q_NBO_) for some key bonds and atoms of the reaction pathway for the first ethylene insertion.

	WBI				Q_NBO_ (*e*)	
	B (Pd-C_1_)	B (C_2_-C_3_)	B (Pd-C_3_)	B (C_1_-C_2_)	Pd	C_1_
CatA + C_2_H_4_	0.704	2.039			0.318	−0.819
cIM1_A_	0.718	1.550	0.408		0.243	−0.768
cTS1_A_	0.406	1.310	0.553	0.538	0.161	−0.739
cPR1_A_	0.010	1.069	0.672	1.108	0.219	−0.666
	B (Pd-C_1_)	B (C_2_-C_3_)	B (Pd-C_2_)	B (C_1_-C_3_)	Pd	C_1_
tIM1_A_	0.743	1.680	0.297		0.194	−0.782
tTS1_A_	0.365	1.296	0.577	0.618	0.210	−0.769
tPR1_A_		0.035	0.727	1.007	0.316	−0.666

**Table 2:** The Wiberg bond indices (WBIs) and natural charges (Q_NBO_) for some key bonds and atoms of the reaction pathway for cyclopropenone.

	WBI					Q_NBO_ (*e*)		
Path A_1a_	B (Pd-C_3_)	B (Pd-C_4_)	B (Pd-C_5_)	B (C_4_-C_5_)	B (C_3_-C_4_)	Pd	C_4_	C_5_
cPR1_A_ + 1a	0.672			1.078		0.219	0.521	−0.016
TS1_A_	0.691	0.181	0.298	0.730	0.025	0.185	0.567	−0.051
2A	0.629	0.577	0.564	0.137	0.092	0.166	0.587	0.021
TS2_A_	0.329	0.263	0.667	0.085	0.554	0.200	0.584	0.018
4A	0.017		0.685	0.061	1.008	0.370	0.583	0.022
Path B_1a_	B (Pd-C_3_)	B (Pd-O_1_)	B (C_4_-O_1_)	B (C_4_-C_5_)	B (Pd-C_5_)	Pd	C_4_	O_1_
cPR1_A_ + 1a	0.672		1.682	1.078		0.219	0.521	−0.594
TS3_A_	0.337	0.507	1.176	1.034		0.340	0.364	−0.596
3A	0.015	0.588	0.958	0.921	0.020	0.415	0.271	−0.686
TS4_A_	0.017	0.557	1.078	0.744	0.148	0.414	0.320	−0.641
4A	0.017	0.394	1.449	0.061	0.685	0.370	0.583	−0.556

**Table 3:** The Wiberg bond indices (WBIs) and natural charges (Q_NBO_) for some key bonds and atoms of the pathway of generation CO from cyclopropenone.

WBI							Q_NBO_ (*e*)			
Path A_CO_	B (C_4_-O_1_)	B (C_4_-C_5_)	B (C_4_-C_6_)	B (C_5_-C_6_)			C_4_	C_5_	C_6_	O_1_
1a	1.682	1.078	1.078	1.478			0.521	−0.016	−0.016	−0.594
TS1_1a_	1.887	1.269	0.566	1.505			0.656	−0.221	0.060	−0.476
IM1_1a_	2.025	1.189	0.415	1.585			0.687	−0.314	0.090	−0.405
TS2_1a_	2.063	0.806	0.391	1.964			0.557	−0.222	0.102	−0.442
CO + C_2_Ph_2_	2.250			2.660			0.506	0.006	0.006	−0.506
Path B_CO_	B (Pd-C_3_)	B (Pd-C_5_)	B (C_3_-C_5_)	B (Pd-C_6_)	B (Pd-C_4_)	B (C_4_-C_6_)	Pd	C_4_	C_6_	O_1_
2A	0.629	0.564	0.136	0.080	0.577	1.109	0.166	0.587	−0.184	−0.499
TS1_AEtCO_	0.316	0.429	0.536	0.117	0.633	1.544	0.136	0.557	−0.146	−0.504
IM1_AEtCO_	0.017	0.206	1.009	0.206	0.650	1.046	0.215	0.352	−0.136	0.016
TS2_AEtCO_	0.012	0.034	1.003	0.404	0.834	0.500	0.089	0.604	−0.171	−0.454
IM2_AEtCO_	0.020	0.031	0.996	0.605	0.908	0.131	0.079	0.669	−0.091	−0.424
TS3_AEtCO_	0.014	0.051	0.998	0.644	0.467	0.018	0.128	0.616	−0.031	−0.475
PR_AEtCO_	0.010	0.050	0.995	0.562			0.273	0.506	−0.051	−0.506
Path B’_CO_	B (Pd-C_3_)	B (Pd-C_4_)	B (Pd-C_5_)	B (C_4_-C_6_)	B (C_5_-C_6_)	B (C_3_-C_5_)	Pd	C_4_	C_6_	O_1_
2A	0.629	0.577	0.564	1.109	1.548	0.136	0.166	0.587	−0.184	−0.499
TS1_ACOEt_	0.675	0.115	0.368	0.619	1.815	0.022	0.155	0.549	−0.023	−0.441
IM1_ACOEt_	0.701		0.200		2.431	0.026	0.205	0.506	−0.024	0.506
TS2_ACOEt_	0.368		0.291		1.872	0.567	0.180		0.006	
PR_AEtCO_	0.010		0.050		1.233	1.245	0.273		−0.051	

**Table 4:** The Wiberg bond indices (WBIs) and natural charges (Q_NBO_) for some key bonds and atoms of the reaction pathway for generation of UnitB.

WBI					Q_NBO_ (*e*)			
CO Insertion	B (Pd-C_11_)	B (Pd-C_9_)	B (C_9_-C_11_)	B (C_11_-O_2_)	Pd	C_11_	O_2_	
PR_AE2_ + CO		0.706		1.138	0.232	0.506	−0.506	
IM_ACO_	0.815	0.701	0.076	2.224	0.026	0.665	−0.415	
TS_ACO_	0.798	0.306	0.668	2.001	0.216	0.571	−0.455	
PR_ACO_	0.748	0.158	0.919	1.921	0.258	0.554	−0.459	
C_2_H_4_ insertion	B (Pd-C_11_)	B (Pd-C_12_)	B (C_12_-C_13_)	B (C_11_-C_13_)	Pd	C_11_	C_12_	C_13_
PR_ACO_	0.748		2.039		0.258	0.554	−0.427	−0.427
IM_ACOE_	0.650	0.411	1.522	0.051	0.187	0.554	−0.490	−0.441
TS_ACOE_	0.259	0.598	1.206	0.618	0.167	0.548	−0.505	−0.546
PR_ACOE_	0.022	0.705	1.010	1.026	0.240	0.652	−0.589	−0.564

**Table 5:** The Wiberg bond indices (WBIs) and natural charges (Q_NBO_) for some key bonds and atoms of the reaction pathway of the allyl acetate (AAc) insertion.

WBI					Q_NBO_ (*e*)			
	B (Pd-C_9_)	B (Pd-C_15_)	B (C_14_-C_15_)	B (C_9_-C_14_)	Pd	C_9_	C_15_	C_14_
PR_AE2_ + AAc	0.706		2.039		0.232	−0.517	−0.427	−0.427
TS_21AAc_	0.406	0.518	1.302	0.487	0.168	−0.465	−0.339	−0.445
PR_21AAc_	0.066	0.663	1.014	1.004	0.244	−0.440	−0.345	−0.489
	B (Pd-C_9_)	B (Pd-C_14_)	B (C_14_-C_15_)	B (C_9_-C_15_)	Pd	C_9_	C_14_	C_15_
PR_AE2_ + AAc	0.706		2.039		0.232	−0.517	−0.427	−0.427
TS_12AAc_	0.382	0.547	1.302	0.486	0.145	−0.473	−0.511	−0.270
PR_12AAc_	0.016	0.689	1.010	0.988	0.222	−0.465	−0.544	−0.305

**Table 6:** The global reactivity index (GRI) and Fukui function values for some molecules.

	*η ^a^*	*μ ^b^*	*ω ^c^*	*N*_Nu_ *^d^*	*f^+^* ^e^	*f^−^* ^f^	
					Pd	C_a_	C_b_
CatA	6.534	−4.175	1.473	3.105	0.228		
C_2_H_4_	13.705	−3.509	0.592	1.865		0.313	0.313
1a	7.360	−4.108	1.303	3.283		0.059	0.057
AAc	11.579	−3.524	0.771	1.972		0.167	0.116

*^a^* Chemical hardness (*η*, in eV). *^b^* Electronic chemical potential (*μ*, in eV). *^c^* Global electrophilicity (*ω*, in eV). *^d^* Global nucleophilicity (*N*_Nu_, in eV). ^e^ Nucleophilic attack Fukui function for Pd (*f^+^*, in e). ^f^ Electrophilic attack Fukui function for C_a_, C_b_ (*f^−^*, in e).

## Data Availability

The data presented in this study are available in the Supplementary Materials.

## Supplementary Materials

The following supporting information can be downloaded at: https://www.mdpi.com/article/10.3390/polym14235273/s1. Table S1: Energies of all the optimized structures. Table S2: Additional computational details and detailed data of Fukui functions values for some molecules [54,55,56,57,58,59,60].

## References

[1] Wang H., Chen C. (2022). Transition metal-catalyzed copolymerization of olefins with polar functional monomers. Comprehensive Organometallic Chemistry IV.

[2] Karimi M., Arabi H., Sadjadi S. (2022). New advances in olefin homo and copolymerization using neutral, single component palladium/nickel complexes ligated by a phosphine-sulfonate. J. Catal..

[3] Jian Z. (2018). Synthesis of functionalized polyolefins: Design from catalysts to polar monomers. Acta Polym. Sin..

[4] Johnson L.K., Killian C.M., Brookhart M. (1995). New Pd(II)- and Ni(II)-based catalysts for polymerization of ethylene and α-Olefins. J. Am. Chem. Soc..

[5] Drent E., van Dijk R., van Ginkel R., van Oort B., Pugh R.I. (2002). Palladium catalysed copolymerisation of ethene with alkylacrylates: Polar comonomer built into the linear polymer chain. Chem. Comm..

[6] Nakamura A., Ito S., Nozaki K. (2009). Coordination-insertion copolymerization of fundamental polar monomers. Chem. Rev..

[7] Friedberger T., Wucher P., Mecking S. (2012). Mechanistic insights into polar monomer insertion polymerization from acrylamides. J. Am. Chem. Soc..

[8] Rünzi T., Fröhlich D., Mecking S. (2010). Direct synthesis of ethylene-acrylic acid copolymers by insertion polymerization. J. Am. Chem. Soc..

[9] Ito S., Munakata K., Nakamura A., Nozaki K. (2009). Copolymerization of vinyl acetate with ethylene by palladium/alkylphosphine-sulfonate catalysts. J. Am. Chem. Soc..

[10] Luo S., Vela J., Lief G.R., Jordan R.F. (2007). Copolymerization of ethylene and alkyl vinyl ethers by a (phosphine-sulfonate)PdMe catalyst. J. Am. Chem. Soc..

[11] Skupov K.M., Piche L., Claverie J.P. (2008). Linear polyethylene with tunable surface properties by catalytic copolymerization of ethylene with N-vinyl-2-pyrrolidinone and N-isopropylacrylamide. Macromolecules.

[12] Daigle J.-C., Piche L., Claverie J.P. (2011). Preparation of functional polyethylenes by catalytic copolymerization. Macromolecules.

[13] Guo L., Gao H., Guan Q., Hu H., Deng J., Liu J., Liu F., Wu Q. (2012). Substituent effects of the backbone in α-diimine palladium catalysts on homo and copolymerization of ethylene with methyl acrylate. Organometallics.

[14] Carrow B.P., Nozaki K. (2012). Synthesis of functional polyolefins using cationic bisphosphine monoxide-palladium complexes. J. Am. Chem. Soc..

[15] Mitsushige Y., Carrow B.P., Ito S., Nozaki K. (2016). Ligand-controlled insertion regioselectivity accelerates copolymerization of ethylene with methyl acrylate by cationic bisphosphine monoxide-palladium catalysts. Chem. Sci..

[16] Allen K.E., Campos J., Daugulis O., Brookhart M. (2015). Living polymerization of ethylene and copolymerization of ethylene/methyl acrylate using “Sandwich” diimine palladium catalysts. ACS Catal..

[17] Sui X., Dai S., Chen C. (2015). Ethylene polymerization and copolymerization with polar monomers by cationic phosphine phosphonic amide palladium complexes. ACS Catal..

[18] Nakano R., Nozaki K. (2015). Copolymerization of propylene and polar monomers using Pd/IzQO catalysts. J. Am. Chem. Soc..

[19] Yasuda H., Nakano R., Ito S., Nozaki K. (2018). Palladium/IzQO-catalyzed coordination-insertion copolymerization of ethylene and 1,1-disubstituted ethylenes bearing a polar functional group. J. Am. Chem. Soc..

[20] Luckham S.L.J., Nozaki K. (2021). Toward the copolymerization of propylene with polar comonomers. Acc. Chem. Res..

[21] Ota Y., Ito S., Kobayashi M., Kitade S., Sakata K., Tayano T., Nozaki K. (2016). Crystalline isotactic polar polypropylene from the palladium-catalyzed copolymerization of propylene and polar monomers. Angew. Chem. Int. Edit..

[22] Zhang W., Waddell P.M., Tiedemann M.A., Padilla C.E., Mei J., Chen L., Carrow B.P. (2018). Electron-rich metal cations enable synthesis of high molecular weight, linear functional polyethylenes. J. Am. Chem. Soc..

[23] Chen M., Chen C. (2018). A versatile ligand platform for palladium- and nickel-catalyzed ethylene copolymerization with polar monomers. Angew. Chem. Int. Edit..

[24] Gao J., Cai W., Hu Y., Chen C. (2019). Improving the flame retardancy of polyethylenes through the palladium-catalyzed incorporation of polar comonomers. Polym. Chem..

[25] Chen M., Chen C. (2019). Direct and tandem routes for the copolymerization of ethylene with polar functionalized internal olefins. Angew. Chem. Int. Edit..

[26] Zou C., Chen C. (2019). Polar-functionalized, crosslinkable, self-healing, and photoreponsive polyolefins. Angew. Chem. Int. Ed..

[27] Wang X., Nozaki K. (2018). Selective chain-end functionalization of polar polyethylenes: Orthogonal reactivity of carbene and polar vinyl monomers in their copolymerization with ethylene. J. Am. Chem. Soc..

[28] Matsuda T., Sakurai Y. (2013). Palladium-catalyzed ring-opening alkynylation of cyclopropenones. Euro. J. Org. Chem..

[29] Fumagalli G., Stanton S., Bower J.F. (2017). Recent methodologies that exploit C-C single-bond cleavage of strained ring systems by transition metal complexes. Chem. Rev..

[30] Wang X., Seidel F.W., Nozaki K. (2019). Synthesis of polyethylene with in-chain α,β-unsaturated ketone and isolated ketone units: Pd-catalyzed ring opening copolymerization of cyclopropenone with ethylene. Angew. Chem. Int. Ed..

[31] Dolbier W.R., Battiste M.A. (2003). Structure, synthesis, and chemical reactions of fluorinated cyclopropanes and cyclopropenes. Chem. Rev..

[32] Luo Y., Shan C., Liu S., Zhang T., Zhu L., Zhong K., Bai R., Lan Y. (2019). Oxidative addition promoted C-C bond cleavage in Rh-mediated cyclopropenone activation: A DFT study. ACS Catal..

[33] Frisch M.J., Trucks G.W., Schlegel H.B., Scuseria G.E., Robb M.A., Cheeseman J.R., Scalmani G., Barone V., Petersson G.A., Nakatsuji H. (2016). Gaussian 16, Revision C.01.

[34] Becke A.D. (1993). Density-functional thermochemistry. III. The role of exact exchange. J. Chem. Phys..

[35] Grimme S., Antony J., Ehrlich S., Krieg H. (2010). A consistent and accurate ab initio parametrization of density functional dispersion correction (DFT-D) for the 94 elements H-Pu. J. Chem. Phys..

[36] Grimme S., Ehrlich S., Goerigk L. (2011). Effect of the damping function in dispersion corrected density functional theory. J. Comput. Chem..

[37] Smith D.G., Burns L.A., Patkowski K., Sherrill C.D. (2016). Revised damping parameters for the D3 dispersion correction to density functional theory. J. Phys. Chem. Lett..

[38] Miehlich B., Savin A., Stoll H., Preuss H. (1989). Results obtained with the correlation energy density functionals of Becke and Lee, Yang and Parr. Chem. Phys. Lett..

[39] Zhao Y., Truhlar D.G. (2008). Density functionals with broad applicability in chemistry. Acc. Chem. Res..

[40] Zhao Y., Truhlar D.G. (2009). Benchmark energetic data in a model system for grubbs II metathesis catalysis and their use for the development, assessment, and validation of electronic structure methods. J. Chem. Theory Comput..

[41] Marenich A.V., Cramer C.J., Truhlar D.G. (2009). Universal solvation model based on solute electron density and on a continuum model of the solvent defined by the bulk dielectric constant and atomic surface tensions. J. Phys. Chem. B.

[42] Reed A.E., Curtiss L.A., Weinhold F. (1988). Intermolecular interactions from a natural bond orbital, donor-acceptor viewpoint. Chem. Rev..

[43] Dang Y., Qu S., Wang Z.-X., Wang X. (2014). A computational mechanistic study of an unprecedented heck-type relay reaction: Insight into the origins of regio- and enantioselectivities. J. Am. Chem. Soc..

[44] Deng L., Fu Y., Lee S.Y., Wang C., Liu P., Dong G. (2019). Kinetic resolution via Rh-catalyzed C-C activation of cyclobutanones at room temperature. J. Am. Chem. Soc..

[45] Li Y., Chen H., Qu L.-B., Houk K.N., Lan Y. (2019). Origin of regiochemical control in Rh(III)/Rh(V)-catalyzed reactions of unsaturated oximes and alkenes to form pyrdines. ACS Catal..

[46] Palani V., Hugelshofer C.L., Kevlishvili I., Liu P., Sarpong R. (2019). A short synthesis of delavatine a unveils new insights into site-selective cross-coupling of 3,5-dibromo-2-pyrone. J. Am. Chem. Soc..

[47] Qi X., Kohler D.G., Hull K.L., Liu P. (2019). Energy decomposition analyses reveal the origins of catalyst and nucleophile effects on regioselectivity in nucleopalladation of alkenes. J. Am. Chem. Soc..

[48] Zhao R., Chen X.Y., Wang Z.X. (2021). Insight into the selective methylene oxidation catalyzed by Mn(CF_3_-PDP)(SbF_6_)_2_/H_2_O_2_/CH_2_ClCO_2_H) system: A DFT mechanistic study. Org. Lett..

[49] Liu Z., Lu Y., Guo J., Hu W., Wang Z.X. (2020). DFT mechanistic account for the site selectivity of electron-rich C(sp3)–H bond in the manganese-catalyzed aminations. Org. Lett..

[50] Ren X., Lu Y., Lu G., Wang Z.X. (2020). Density functional theory mechanistic study of Ni-catalyzed reductive alkyne–alkyne cyclodimerization: Oxidative cyclization versus outer-sphere proton transfer. Org. Lett..

[51] Yang Y.-F., Chen G., Hong X., Yu J.-Q., Houk K.N. (2017). The origins of dramatic differences in five-membered vs six-membered chelation of Pd(II) on efficiency of C(sp3)-H bond activation. J. Am. Chem. Soc..

[52] Yu S.-Y., Peng X., Wang F., Cao J., Wang F., Zhang C.-G. (2022). Density functional theory study of the regioselectivity in copolymerization of bis-styrenic molecules with propylene using zirconocene catalyst. Catalysts.

[53] Lu T., Chen F.-W. (2021). Interaction region indicator (IRI): A simple real space function clearly revealing both chemical bonds and weak interactions. Chem.-Methods.

[54] Lu T., Chen Q.X. (2022). Realization of conceptual density functional theory and information-theoretic approach in Multiwfn program. Conceptual Density Functional Theory.

[55] Parr R.G., Pearson R.G. (1983). Absolute hardness-companion parameter to absolute electronegativity. J. Am. Chem. Soc..

[56] Parr R.G., Von Szentpaly L., Liu S.B. (1999). Electrophilicity index. J. Am. Chem. Soc..

[57] Domingo L.R., Chamorro E., Pérez P. (2008). Understanding the reactivity of captodative ethylenes in polar cycloaddition reactions. A theoretical study. J. Org. Chem..

[58] Parr R.G., Yang W. (1984). Density functional approach to the frontier-electron theory of chemical reactivity. J. Am. Chem. Soc..

[59] Fu R., Lu T., Chen F.-W. (2014). Comparing methods for predicting the reactive site of electrophilic substitution. Acta Phys.-Chim. Sin..

[60] Lu T., Chen F.-W. (2012). Multiwfn: A multifunctional wavefunction analyzer. J. Comput. Chem..

[61] Humphrey W., Dalke A., Schulten K. (1996). VMD: Visual molecular dynamics. J. Mol. Graphics.

[62] Rezabal E., Ugalde J.M., Frenking G. (2017). The trans effect in palladium phosphine sulfonate complexes. J. Phys. Chem. A.

[63] Sun J., Chen M., Luo G., Chen C., Luo Y. (2019). Diphosphazane-monoxide and phosphine-sulfonate palladium catalyzed ethylene copolymerization with polar monomers: A computational study. Organometallics.

[64] Noda S., Nakamura A., Kochi T., Chung L.W., Morokuma K., Nozaki K. (2009). Mechanistic studies on the formation of linear polyethylene chain catalyzed by palladium phosphine-sulfonate complexes: Experiment and theoretical studies. J. Am. Chem. Soc..

[65] Kuzmanich G., Gard M.N., Garcia-Garibay M.A. (2009). Photonic amplification by a singlet-state quantum chain reaction in the photodecarbonylation of crystalline diarylcyclopropenones. J. Am. Chem. Soc..

[66] Poloukhtine A., Popik V.V. (2006). Mechanism of the cyclopropenone decarbonylation reaction. A density functional theory and transient spectroscopy study. J. Phys. Chem. A.

