# Supplementary material for: Density Functional Theory Analysis of the Copolymerization of Cyclopropenone with Ethylene Using a Palladium Catalyst
Source: Polymers (Basel). 2022 Dec 2;14(23):5273. doi: 10.3390/polym14235273 (PMC9739415; doi:10.3390/polym14235273)
Supplement: Supplementary file 1 [file polymers-14-05273-s001.zip › polymers-2062249-SI.pdf]

# Supplementary Materials

## Density Functional Theory Analysis of the Copolymerization of Cyclopropenone with Ethylene Using a Palladium Catalyst

**Chenggen Zhang [1,*] , Shuyuan Yu [1,2,*] , Fei Wang [1], Fuping Wang [1], Jian Cao [1] , Huimin Zheng [1] , Xiaoyu Chen [1] and Aijin Ren [1]**

[1] College of Chemistry and Material Science, Langfang Normal University, Langfang 065000, China

[2] College of Chemistry and Chemical Engineering, University of Chinese Academy of Sciences, Beijing 100049, China

***** Correspondence: chgzhang04@mails.ucas.ac.cn (C.Z.); yushuyuan05@mails.ucas.ac.cn (S.Y.)

**Contents**

**Table S1**: Energies of all the optimized structures.
**Table S2**: Additional computational details and detailed data of Fukui functions values for some molecules.

**Table S1: Energies of all the structures.**

Gibbs free energies (G) (in *a.u.*) of all the structures for the main text

| | | | | | |
|---|---|---|---|---|---|
| CatA | $C_2H_4$ | $cIM1_A$ | $cTS1_A$ | $cPR1_A$ | $tIM1_A$ |
| -2055.81925 | -78.53930 | -2134.356108 | -2134.335544 | -2134.383986 | -2134.36715 |
| $tTS1_A$ | $tPR1_A$ | | | | |
| -2134.323624 | -2134.35961 | | | | |
| 1a | $TS1_A$ | 2A | $TS2_A$ | 4A | $TS3_A$ |
| -652.54106 | -2786.88886 | -2786.90273 | -2786.88677 | -2786.96061 | -2786.85778 |
| 3A | $TS4_A$ | | | | |
| -2786.90367 | -2786.89978 | | | | |
| $IM_{AE1}$ | $TS_{AE1}$ | $PR_{AE1}$ | $IM_{AE2}$ | $TS_{AE2}$ | $PR_{AE2}$ |
| -2865.49355 | -2865.47984 | -2865.50651 | -2944.04813 | -2944.02047 | -2944.06330 |
| $TS1_{1a}$ | $IM1_{1a}$ | $TS2_{1a}$ | CO | $C_2Ph_2$ | |
| -652.48383 | -652.48756 | -652.48584 | -113.32316 | -539.23344 | |
| $TS1_{AEtCO}$ | $IM1_{AEtCO}$ | $TS2_{AEtCO}$ | $IM2_{AEtCO}$ | $TS3_{AEtCO}$ | $PR_{AEtCO}$ |
| -2786.88988 | -2786.96727 | -2786.95583 | -2786.96417 | -2786.94115 | -2673.62738 |
| $TS1_{ACOEt}$ | $IM1_{ACOEt}$ | $TS2_{ACOEt}$ | | | |
| -2786.84846 | -2673.60987 | -2673.56727 | | | |
| $IM_{ACO}$ | $TS_{ACO}$ | $PR_{ACO}$ | $IM_{ACOE}$ | $TS_{ACOE}$ | $PR_{ACOE}$ |
| -3057.39288 | -3057.37072 | -3057.37892 | -3135.93311 | -3135.91842 | -3135.95968 |
| $TS_{21AAc}$ | $PR_{21AAc}$ | $TS_{12AAc}$ | $PR_{12AAc}$ | | |
| -3289.68602 | -3289.73392 | -3289.68655 | -3289.73224 | | |

**Table S2**: Additional computational details and detailed data of Fukui functions values for some molecules.

## (A) Additional computational details:

The global reactivity index and Fukui functions values were performed using the Multiwfn program [1,2]. The calculations also used a mixed basis set (SDD for Pd and 6-31G(d) for other non-metal atoms).

Vertical ionization potential: $\mathrm{VIP} = E_{N-1} - E_N$ ,

Vertical electron affinity: $\mathrm{VEA} = E_\mathrm{N} - E_{N-1}$,

where N denotes the number of electrons for a stable system.

Mulliken electronegativity: $\chi = \dfrac{\mathrm{VIP} + \mathrm{VEA}}{2}$ ,

Chemical potential: $\mu = -\chi$ ,

Chemical hardness: $\eta = \mathrm{VIP} - \mathrm{VEA}$ [3],

Electrophilicity index [4]: $\omega = \dfrac{\mu^2}{2\eta}$ ,

Nucleophilicity index: $N_{Nu} = E_{HOMO}(Nu) - E_{HOMO}(TCE)$ , where Nu refers to nucleophile and TCE denotes tetracyanoethylene [5].

The Fukui function is defined as follows [6,7], where N denotes the number of electrons, the term $\upsilon$ is external potential.

$$f(\mathrm{r}) = [\frac{\partial \rho(\mathrm{r})}{\partial N}]_\upsilon$$

Nucleophilic attack: $f^+(r) = \rho_{N+1}(r) - \rho_N(r)$ ,

Electrophilic attack: $f^-(r) = \rho_N(r) - \rho_{N-1}(r)$ .

## (B) Detailed data of Fukui functions values for some molecules

| CatA | | | | 1a | | $C_2H_4$ | |
|---|---|---|---|---|---|---|---|
| Atom | $f^+$ | Atom | $f^+$ | Atom | $f^-$ | Atom | $f^-$ |
| 1(P ) | 0.073 | 26(H ) | 0.0159 | 1(C ) | 0.0586 | 1(C ) | 0.3128 |
| 2(C ) | -0.0003 | 27(H ) | 0.0136 | 2(C ) | 0.0565 | 2(H ) | 0.0936 |
| 3(C ) | 0.0036 | 28(H ) | 0.0204 | 3(C ) | 0.0586 | 3(H ) | 0.0936 |
| 4(S ) | 0.0151 | 29(C ) | -0.0048 | 4(O ) | 0.1881 | 4(C ) | 0.3128 |
| 5(O ) | 0.0148 | 30(C ) | 0.0067 | 5(C ) | 0.0248 | 5(H ) | 0.0936 |
| 6(O ) | 0.0115 | 31(C ) | 0.0009 | 6(C ) | 0.0623 | 6(H ) | 0.0936 |
| 7(O ) | 0.0329 | 32(C ) | 0.0172 | 7(C ) | 0.0291 | | |
| 8(C ) | 0.016 | 33(C ) | 0.0134 | 8(C ) | 0.0303 | | |
| 9(C ) | 0.0284 | 34(H ) | 0.0048 | 9(C ) | 0.0328 | AAc | |
| 10(C ) | 0.0219 | 35(C ) | 0.0241 | 10(C ) | 0.0341 | Atom | $f^-$ |
| 11(C ) | 0.012 | 36(H ) | 0.0142 | 11(H ) | 0.0135 | 1(C ) | 0.1160 |
| 12(H ) | 0.0123 | 37(H ) | 0.0127 | 12(H ) | 0.014 | 2(C ) | 0.1670 |

| | | | | | | | | | | |
|---|---|---|---|---|---|---|---|---|---|---|
| 13(H ) | 0.0187 | | 38(H ) | 0.017 | | 13(H ) | 0.0242 | | 3(H ) | 0.0507 |
| 14(H ) | 0.017 | | 39(O ) | -0.0041 | | 14(H ) | 0.0243 | | 4(H ) | 0.0570 |
| 15(H ) | 0.0071 | | 40(O ) | -0.0015 | | 15(H ) | 0.0296 | | 5(C ) | 0.0244 |
| 16(C ) | 0.0724 | | 41(C ) | 0.0049 | | 16(C ) | 0.0248 | | 6(H ) | 0.0379 |
| 17(H ) | 0.0499 | | 42(C ) | 0.0052 | | 17(C ) | 0.0291 | | 7(H ) | 0.026 |
| 18(H ) | 0.0296 | | 43(H ) | 0.0102 | | 18(C ) | 0.0341 | | 8(O ) | 0.0593 |
| 19(C ) | -0.0027 | | 44(H ) | 0.0056 | | 19(C ) | 0.0623 | | 9(C ) | 0.0638 |
| 20(C ) | 0.0148 | | 45(H ) | 0.0052 | | 20(C ) | 0.0328 | | 10(O ) | 0.2079 |
| 21(C ) | 0.0082 | | 46(H ) | 0.0055 | | 21(C ) | 0.0304 | | 11(C ) | 0.0444 |
| 22(C ) | 0.0189 | | 47(H ) | 0.0069 | | 22(H ) | 0.0135 | | 12(H ) | 0.0348 |
| 23(H ) | 0.0065 | | 48(H ) | 0.0114 | | 23(H ) | 0.0243 | | 13(H ) | 0.0363 |
| 24(C ) | 0.0162 | | 49(H ) | 0.0327 | | 24(H ) | 0.0296 | | 14(H ) | 0.0302 |
| 25(C ) | 0.0349 | | 50(Pd) | 0.2283 | | 25(H ) | 0.0242 | | 15(H ) | 0.0442 |
| | | | | | | 26(H ) | 0.014 | | | |

## References


1. Lu, T.; Chen, F.-W. Multiwfn: A multifunctional wavefunction analyzer. *J. Comput. Chem.* **2012**, *33*, 580–592.
2. Lu, T.; Chen, Q.X. Realization of conceptual density functional theory and information-theoretic approach in Multiwfn program. In *Conceptual Density Functional Theory*; WILEY-VCH GmbH: Weinheim, Germany, 2022; pp. 631–647. https://doi.org/10.1002/9783527829941.ch31.
3. Parr, R.G. ; Pearson, R.G. Absolute hardness-companion parameter to absolute electronegativity. *J. Am. Chem. Soc.* **1983**, *105*, 7512–7516.
4. Parr, R.G.; Von Szentpaly, L.; Liu, S.B. Electrophilicity index. *J. Am. Chem. Soc.* **1999**, *121*, 1922–1924.
5. Domingo, L.R.; Chamorro, E.; Pérez, P. Understanding the reactivity of captodative ethylenes in polar cycloaddition reactions. a theoretical study. *J. Org. Chem.* **2008**, *73*, 4615–4624.
6. Parr, R.G.; Yang, W. Density functional approach to the frontier-electron theory of chemical reactivity. *J. Am. Chem. Soc.* **1984**, *106*, 4049–4050.
7. Fu, R.; Lu, T.; Chen, F.-W. Comparing methods for predicting the reactive site of electrophilic substitution. *Acta Phys.-Chim. Sin.* **2014**, *30*, 628–639.
